# Application of Wharton jelly-derived mesenchymal stem cells in patients with pulmonary fibrosis

**DOI:** 10.1186/s13287-022-02746-x

**Published:** 2022-02-15

**Authors:** Mahshid Saleh, Seyedeh Zahra Fotook Kiaei, Maria Kavianpour

**Affiliations:** 1 Department of Applied Cell Sciences, School of Advanced Technologies in Medicine, Tehran University of Medical Sciences, Tehran, Iran; 2 Department of Pulmonary and Critical Care, Shariati Hospital, Tehran University of Medical Sciences, Tehran, Iran

**Keywords:** Stem cell, WJ-MSC, Pulmonary fibrosis, Cell-based therapy

## Abstract

Pulmonary fibrosis is a devastating disease that eventually leads to death and respiratory failure. Despite the wide range of drugs, including corticosteroids, endothelin antagonist, and pirfenidone, there is no effective treatment, and the only main goal of treatment is to alleviate the symptoms as much as possible to slow down the progression of the disease and improve the quality of life. Lung transplantation may be a treatment option for a few people if pulmonary fibrosis develops and there is no established treatment. Pulmonary fibrosis caused by the COVID19 virus is another problem that we face in most patients despite the efforts of the international medical communities. Therefore, achieving alternative treatment for patients is a great success. Today, basic research using stem cells on pulmonary fibrosis has published promising results. New stem cell-based therapies can be helpful in patients with pulmonary fibrosis. Wharton jelly-derived mesenchymal stem cells are easily isolated in large quantities and made available for clinical trials without causing ethical problems. These cells have higher flexibility and proliferation potential than other cells isolated from different sources and differentiated into various cells in laboratory environments. More clinical trials are needed to determine the safety and efficacy of these cells. This study will investigate the cellular and molecular mechanisms and possible effects of Wharton jelly-derived mesenchymal stem cells in pulmonary fibrosis.

## Introduction

The lung organ is located in the chest, where the delicate lung tissue is protected by the bony and muscular cage of the chest. This tissue provides a constant flow of oxygen to the human body's tissues, and in the meantime, the blood is cleared of carbon dioxide. Air is regularly pumped in and out through air ducts. The airways are divided into upper and lower airway systems. The transmission between the two air systems is located above the larynx [1]. Tissue fibrosis and its failure are significantly associated with mortality worldwide [2]. Fibrosis is defined by excessive deposition of connective tissue components, including myofibroblasts [3]. Fibrotic disease is usually characterized by a progressive and incomplete cycle of abnormally high accumulation of myofibroblasts [2]. It can affect almost all tissues. In this disease process, the affected organs lose their physiological function and become defective [3]. When the injury occurs, tissue microenvironmental regeneration is critical to restoring normal organ function. Inflammation and subsequent acute inflammatory reactions for various reasons, including infection or injury, can disrupt epithelial and endothelial integrity and lead to complications such as edema, leukocyte uptake, and angiogenesis. Regeneration of damaged tissue and elimination of inflammation through apoptotic and phagocytic pathways often leaves minimal damage. However, the presence of a persistent inflammation can cause idiopathic pulmonary fibrosis (IPF) [4, 5].

### Pulmonary fibrosis (PF)

Pulmonary fibrosis is characterized by damage to the alveolar epithelial cells, regeneration of lung tissue, an unusual accumulation of extracellular matrix, and fibroblasts in the tissue [6, 7], which leads to respiratory failure and death [8]. When inflammation predominates, a pathogenic fibrotic reaction occurs, during which angiogenic, proinflammatory, fibrosis-causing cytokines, destructive enzymes, and growth factors accumulate at the injury site [4, 9]. The most common type of pulmonary fibrosis, idiopathic pulmonary fibrosis (IPF), is a progressive disease of the lower respiratory tract with a 5-year survival rate that usually affects adults over 40 years of age [10, 11]. The factors involved in the onset of histopathological cascade in pulmonary fibrosis (PF) are unknown [12, 13]. PF-related risk factors in three categories of comorbidities, internal and external risk factors, are listed in Table 1 [14–18].

**Table 1:** Risk factors in pulmonary fibrosis

External risk factors	Internal risk factors	Co-morbidities
Cigarette smoking	Genetics	Gastroesophageal reflux
Environmental pollutants	Aging	Obstructive sleep apnea
Air pollution	Sex	Diabetes mellitus
Drugs	Lung microbiome	Virus infection
Certain occupations		Chronic aspiration
Cancer treatments (radiation treatments)		

### Pathophysiology of pulmonary fibrosis

Pulmonary fibrosis has resulted from recurrent damage to lung tissue leading to epithelial damage followed by the destruction of the alveolar-capillary basement membrane. This process causes fibroblast cells to infiltrate and myofibroblasts to become active. Eventually, the lung tissue loses its function and progresses to death [13]. Studies have shown that alveolar epithelial cell damage is an essential factor in the pathogenesis of idiopathic pulmonary fibrosis. Studies have reported type 2 alveolar cell (ATII) hyperplasia in patients with IPF [19]. In familial forms of pulmonary fibrosis, mutations in genes involved in tissue regeneration lead to damage or apoptosis of ATII cells [20]. ATII cells damage leads to ineffective reconstitution of normal epithelium and fibrosis development with activation of myofibroblasts [21, 22]. Repeated damage to the alveolar region creates a pro-inflammatory environment [2]. Inflammation leads to an abnormal wound healing response explained by genetic changes in crucial genes such as TGFb1, tumor necrosis factor-alpha (TNFα), MCP1/CCL2, MIP1a/CCL3, and surfactant protein C (SFTPC) [2, 23, 24].

TNF-α is a pleiotropic cytokine produced by a variety of cells in response to infection or damage. Improper secretion of TNF is involved in the pathogenesis of various human diseases, including infection, transplant rejection, cancer, inflammatory diseases, and pulmonary fibrosis [25, 26]. This cytokine plays an essential role in cell adhesive, inflammatory responses, migration, and activation of cytokine and chemokine cascades [27].

One of the most substantial profibrotic factors is TGF-b, which promotes lung fibrosis by using and activating monocytes and fibroblasts and producing an extracellular matrix. TGF-b1 induces fibroblast proliferation by inducing fibroblast growth factor 2 and subsequent activation of the MAPK signaling pathway [28], leading these cells to differentiate into myofibroblasts. TGF-b promotes ECM production by promoting ECM gene transcription [29]. The proinflammatory chemokines MCP1 / CCL2 and MIP1a / CCL3 are among the monocyte invoking chemokines [30]. Macrophages and fibroblasts express CCL2 / MCP-1, and its production is required for pulmonary fibrosis [31]. CCL3 / MIP-1a also helps to aggravate lung damage [32]. Studies have shown that in the BAL secretions of patients with IPF, the levels of CCL2 / MCP-1 and CCL3 / MIP-1a were increased compared to healthy individuals [33, 34].

### Wharton jelly-derived mesenchymal stem cells

Mesenchymal stem cells are multipotent progenitor cells that can proliferate and regenerate [35]. Mesenchymal cells, like ready-made soldiers, are found in all types of adult tissues, including bone marrow, fat, skin, placenta, and heart, which migrate easily through the blood vessels when damaged by the secretion of various inflammatory factors and the invocation of inflammatory cells. Due to its surface receptors, it implants with SDF1 factors secreted from the affected area and controls the immune system by secreting various factors [36]. Rich sources of MSCs include tissues such as the placenta, umbilical cord, amniotic fluid, and amniotic membrane, considered medical waste [37]. Compared to adult tissues, stem cells isolated from the amniotic membrane (AM), chorionic plate (CP), peritoneal, and umbilical cord (UC) tissues have more advantages [38–40]. Umbilical Cord consists of two arteries and a vein inserted into a particular mucosal connective tissue known as Wharton jelly (WJ), which is covered by the amniotic epithelium. UC-MSC have a distinct capacity for self-renewal and the ability to differentiate into adipocytes, osteocytes, chondrocytes, neurons, and liver cells. In addition, when cells enter the host body, they accumulate in damaged tissue or inflamed areas and accelerate tissue repair by modulating the immune system [41, 42]. Wharton Jelly isolated from mesenchymal stem cells (hWJMSC) have been described as the best source of MSC [43]. The findings clearly show that WJ-MSC can be the best suggestion for clinical use due to its advantages such as higher proliferation and differentiation potential, easy access, easy and noninvasive separation, a large number of cells, and no ethical problems [44, 45]. Age is an essential issue in cells isolated from the donor [46]. Young donor cells in the culture medium are less exposed to damage and oxidative changes, age much more slowly and have a higher proliferation rate than the older donor [47, 48]. Because of such benefits, Wharton jelly-derived stem cells have received significant attention in various diseases. Mesenchymal stem cells secrete trophic factors that maintain cell survival, including stromal derived factor-1 (SDF-1), hepatocyte growth factor (HGF), and insulin-like growth factor (IGF-1), epithelial growth factor (EGF), nerve growth factor (NGF), transforming growth factor-alpha (TGF-a), and tissue angiogenesis vascular endothelial growth factor (VEGF)[49]. Also, the culture medium of WJ-MSCs contains several secretory factors such as RANTES, MCP-1, MIP-1, IL-12, IL-15, IL-6, IL-8, IL-2, and PGE2. Its immunomodulatory effects mediate through these factors [50].

### Immunomodulatory effects of Wharton jelly-derived mesenchymal stem cells

According to preclinical and clinical studies performed on hWJMSC, these cells seem to be an excellent source in cell-based reconstructive medicine and clinical trials. The WJ-MSC can also suppress the immune system and modulate the immune system through cell-to-cell contact and secretion of soluble factors, thus placing them as suitable candidates for cell therapy in allogeneic transplantation [51]. WJ-MSCs show minimal human leukocyte antigen (HLA) class I antigen expression and no HLA-DR. [52, 53]. HLA-G6 is a type of immunosuppressant produced by WJ-MSCs that inhibits the cytolytic activity of NK cells and does not express the markers CD40, CD80, or CD86 involved in T cell activation [42]. HLA- G6 is produced by trophoblasts and protects the fetus from immune-based degradation [54]. IL-6 (interleukin-6) secreted by mesenchymal stem cells isolated from the umbilical cord leads dendritic cells to tolerant phenotypes [55]. Factors such as TGF-β1, IL-10 (interleukin 10), HGF (Hepatocyte growth factor), PGE2 (prostaglandin E2), IDO (indolamine 2, 3-dioxygenase), galactin-1 are secreted by mesenchymal stem cells. They play an essential role in regulating the immune system [42, 56]. In addition, studies have shown increased expression of anti-inflammatory molecules such as CD200 or PD-L1 (programmed death ligand 1) in WJ-MSC mesenchymal stem cells [57].

MSC-derived exosomes prevent inflammatory cells from penetrating the injury site, reduce lung damage, reduce inflammatory cytokines such as TNF-α and IL-6, and multiple paracrine factors, including TNF, Angiopoietin-1, TSG-6, lipocalin-2, microRNAs, LL-37, and KGF improve survival [58–64]. Mesenchymal stem cells express the TSG-6 gene, which mediates the regulation of immune inflammation [65]. TSG-6 is another major factor that plays a vital role in the tissue repair activity of human mesenchymal stem cells, proven in models of myocardial infarction, peritonitis, and acute corneal and lung damage in mice [66, 67]. TSG-6 cleaves the binding of CXCL8 to heparin by interacting with the GAG-CXCL8 binding site to inhibit the delayed chemotactic effect of neutrophils mediated by CXCL8. In addition, TSG-6 can prevent the migration of leukocytes (mainly neutrophils and macrophages) to the site of inflammation [68]. During tissue damage and the formation of the primary inflammatory phase, proinflammatory macrophages (M1) are activated, clearing pathogenic microorganisms and forming inflammation through extracellular matrix metalloproteases (MMPs) and proinflammatory cytokines [69]. Increased anti-inflammatory M2 macrophages inhibit the inflammatory response produced by chemokines, MMPs, tissue metalloproteinase inhibitors (TIMPs), and fibronectin, leading to the progression of fibrotic lung disease [70–72]. MSCs can alter macrophage phenotypes from an inflammatory M1 phenotype to a more immunomodulatory M2 phenotype, thereby modulating macrophages [73, 74]. On the other hand, mesenchymal stem cells can increase IL-10 levels as an anti-inflammatory protein that activates regulatory cells such as Tregs [75]. Thus, mesenchymal stem cells play a significant role in immune homeostasis by interacting with cytokines, chemokines, and cell surface molecules.

### Mechanism of action of WJ-MSC on pulmonary fibrosis

The exact mechanisms by which stem cells positively affect lung fibroids are not yet known. These mechanisms include several biological pathways, including targeted transplantation into affected areas, differentiation of stem cells into lung epithelial cells, the ability of the immune system to modulate, secretion of anti-inflammatory and anti-fibrotic factors, and the ability of the lung to repair endogenously [76]. Fibroblast activation processes through the epithelium and unfavorable response to anti-inflammatory medications are involved in developing idiopathic pulmonary fibrosis [77]. This process is associated with immune responses including the PGE2 pathway and epithelial mesenchymal transport (EMT) regulators of WISP-1 and BMP4, which are involved in the differentiation of fibroblasts and collagen [78–80]. PGE2 is one of the TGFb-dependent fibrosis antagonists [81]. PGE2 pathways can prevent Fas L apoptosis in fibroblasts and myofibroblasts [82]. Studies show that due to the low concentration of PGE2 in the BAL secretions of pulmonary fibrosis, the physiological antifibrotic action of PGE2 is limited [83]. The cause of this decrease is not well understood [84]. However, low concentrations of PGE2 make type II alveolar cells susceptible to apoptosis [82]. Because WJ-MSC cells express high levels of immune response inhibitors, including IDO and PGE2 [54], they can be used as an alternative treatment to improve pulmonary fibrosis. Studies have highlighted the prominent role of innate and adaptive immune cell populations in the fate of myofibroblasts and fibrosis-related responses in many fibrotic diseases [85, 86]. Immune cells, including macrophages, neutrophils, NK cell, T cells, and B cells, accumulate and become active at the site of inflammation and regulate the improvement and exacerbation of the fibrosis process in various fibrotic organs through various molecular mechanisms [87]. Inflammatory macrophages activate Th2 cells in lung tissue, and this inequality in the Th1 / Th2 immune response is essential in the pathogenesis of pulmonary fibrosis [88]. Macrophages also produce an effective source of fibrotic cytokines, including TGF-b1 and PDGF, chemokines, and proteases [89]. TGF-β signaling is also involved in healthy lung growth and repair of lung damage [90, 91]. This factor can induce fibroblast proliferation, differentiation, migration, and production and contraction of the extracellular matrix. In the adult lung, TGF-β-mediated overexpression of Smad3 plays a vital role in the development of extensive fibrosis [92]. In a study in patients with CTD-IP (Interstitial pneumonia in connective tissue diseases), they reported a significant increase in TGF-β1 levels with a decrease in Tregs and a decrease in the ratio of TGF-β1 to IL-6, indicating an increase in endogenous TGF-β1 which is secreted by immune cells in response to the inflammatory microenvironment. Endogenous TGF-β1 cannot differentiate Tregs due to excessive IL-6 secretion and leads to an imbalance between IL-6 and TGF-β1 in local and systemic modulation of the immune response, resulting in TGF-β1 signaling disrupts. This study examined the paradox of the TGF-β1 pro-fibrotic factor in stimulating α-SMA expression and myofibroblast differentiation and showed that excessive TGF-β1 secretion from MSC cells increased IP-10 levels in UIP-HLF (Usual interstitial pneumonia- human primary lung fibroblasts) and the simultaneous decrease in α-SMA expression (93). Thus, high levels of TGF-β1 secretion by HBMSCs may be an essential mechanism involved in the therapeutic effects of MSCs in promoting the spread of Tregs in IPF patients [94, 95].

**Fig. 1:**
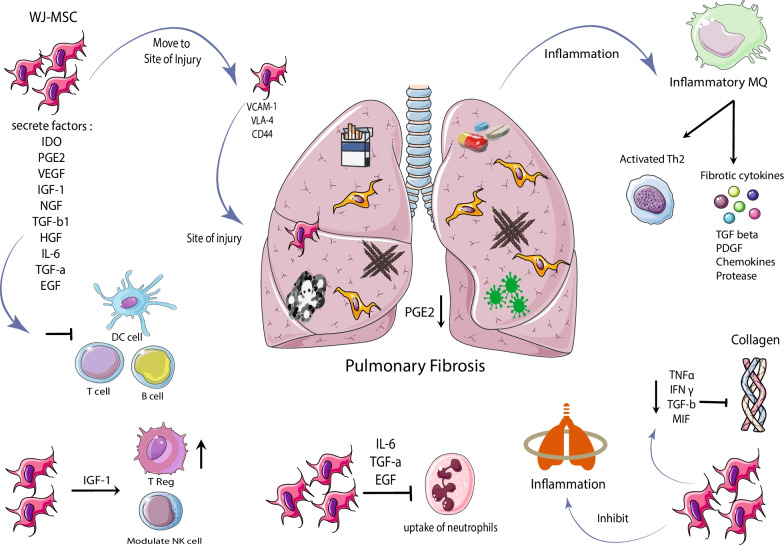
Possible mechanism of Wharton jelly-derived mesenchymal stem cells in pulmonary fibrosis

Mesenchymal stem cells migrate to the injury site through adhesion molecules such as CXCR4, VLA-4, and CD44 [96–98] and secrete soluble factors that inhibit T cells' maturation dendritic cells, reducing activation and proliferation. B cells and inhibition of cytotoxicity Natural killer cells regulate the adaptive and innate immune system [99–101]. Mesenchymal stem cells modulate macrophage phenotypes by diminishing profibrotic macrophage cells and inducing anti-fibrotic effects [102, 103]. WJ-MSCs prevent the uptake of neutrophils into the site of inflammation by IL-6, EGF, and TGF-α. These cells can also modulate NK cells and increase the Treg population through high levels of IGF-1 and its binding proteins [104–107]. A study in an animal model of lung damage showed that WJ-MSCs reduced inflammation and prevented the expression of TNF-α, interferon-γ, growth factor-β, MIF, and significantly reduced collagen [108]. Studies have shown the differentiation of MSCs into lung epithelial cells in a mouse model transplanted with amniotic-derived stem cells, and the CXCR4 / SDF1 axis is thought to be involved [109]. The possible mechanism of Wharton jelly-derived mesenchymal stem cells in pulmonary fibrosis is shown in Fig. 1.

### Mesenchymal stem cells in animal models of pulmonary fibrosis and limitations on their use in pulmonary fibrosis

Lately, adult stem cells, including mesenchymal stem cells (MSCs), have been used in preclinical and clinical trials studies due to their multilineage differentiation, safety, ability to migrate to damaged and inflamed tissue, and immunoregulatory effect in regenerative medicine and cell therapy in respiratory disease are desirable [110–112]. Numerous preclinical studies on animal models of pulmonary fibrosis have shown that mesenchymal stem cells can reduce inflammation, decrease fibrosis, and increase the survival rate [99, 113, 114]. A study by Moroncini et al. used hUC-MSC cells for intravenous injection in C57BL / 6 mice. This study showed that intravenous injection of two doses of MSC significantly decreased fibrosis and inflammation induced by bleomycin through inhibiting IL6-IL10-TGFβ factors, including a reduction in the set of lung M2 macrophages. UC-MSC also showed vigorous anti-fibrotic activity in vivo in the mouse model [115]. Another study showed that transplantation of MenSCs in a mouse model of pulmonary fibrosis significantly improves pulmonary fibrosis by assessing pathological lesions, collagen deposition, and inflammation. These cells also inhibit the apoptosis of MLE-12 cells by suppressing the expression of inflammatory cytokines [116]. A study on the IPF model showed that WJ-derived cells diminished AKT and MMP-2 activation [114]. Another group reported that WJ-MSC represses inflammation, diminishes myofibroblast action, and enhances MMP-9 and TLR-4 receptor expression, leading to the refusal of fibrosis [117]. Because disruption of aging-related pulmonary repair tools is likely to carry the pathogenesis of IPF, the use of younger mesenchymal stem cells may have certain benefits over other sources in the treatment of BLM-induced IPF in older patient's mice [114]. The applicability of WJ-derived MSC as an anti-fibrotic in the lung has been demonstrated in these studies.

Also, in a study by Thashiro and colleagues, they examined differences between adipose mesenchymal stem cells, young and old donors, in older mice after BLM injection. This study showed that young adipose mesenchymal stem cells reduced pro-inflammatory factors, fibrosis, MMP-2 activity, oxidative stress, and apoptotic markers, but MSC treatment of older donors in BLM mice showed associated markers and fibrosis. This model did not decrease [118]. These results suggest that these cells have age-dependent anti-fibrotic properties. It can be said that the age of donors in pulmonary fibrosis is an important issue that should be considered in cell-based clinical trials in IPF patients [76]. Therefore, the use of Wharton jelly-derived mesenchymal stem cells as a young source can provide promising results in patients with pulmonary fibrosis. In the meta-analysis article on animal models of pulmonary fibrosis, 1120 articles were included. In this study, the survival rate, the effect of MSC cells on different models of pulmonary fibrosis, type of MSC, injection route, and injection time were investigated. This study showed that MSC therapy is a safe and effective technique that can significantly ameliorate the survival and pulmonary fibrosis of animal models of pulmonary fibrosis, and this study is the basis for further clinical studies [119].

Today, animal models are used for therapeutic interventions in various diseases. However, these models face limitations. One of the most widely used models for studying pulmonary fibrosis is the BLM model. This animal model is considered an IPF characteristic due to its remarkable resemblance to human idiopathic fibrosis [120, 121]. However, the BLM model does not cover all aspects of human disease, mainly due to the progression of fibrosis; the ability to transfer preclinical data to clinical trials is limited [76]. Although the lungs of patients with IPF are in some cases resemble the fibrotic lungs of these animals, they are not precisely equivalent to humans in the current veterinary classification of fibrotic lung disease, they are not precisely equivalent to humans in the current veterinary classification of fibrotic lung disease [122]. In addition, most therapeutic compounds have been tested on young animals, while studies have clearly shown that older mice are more prone to pulmonary fibrotic damage than younger mice [123].

Regarding efficacy, it can be said that the preclinical efficacy of most anti-fibrotic agents, which are usually tested in the BLM-induced model, is not clinically relevant based on histological examination. Also, the majority of cases in animal studies are not blind interventions for investigators [122]. Also, cases such as the non-reproducibility of experiments between different laboratories and the size of selected animals to produce robust and clinically generalizable data can undermine the validity of experimental studies [122]. Therefore, designing clinical trial studies can help improve the quality of pulmonary fibrosis studies in these patients in the future.

### Clinical trial in the field of cell therapy using mesenchymal stem cells in pulmonary fibrosis

Currently, different cells are used worldwide, including MSCs of allogeneic and autologous origin, from various adipose tissues, placenta, umbilical cord, Wharton jelly, dental pulp and menstrual, NK cell, and Tcell [124]. The safety and efficacy of mesenchymal stem cells to reduce inflammatory lung disease have been shown in animal models [125]. All reports showed that stem cell injections were safe in human clinical trials. Although the effects of cell therapy are not uniform, in some studies, its positive effects have been expressed, and in other studies, these effects have not been observed [126]. The primary purpose of most of these studies is to determine the safety, feasibility, and tolerability of injected mesenchymal stem cells in patients. In a phase 1 study in patients with idiopathic pulmonary fibrosis, adipose-derived stromal cells were used. In this study, ADSCs cells were isolated autologously and injected intrabronchially at 0.5 × 106 kg body weight. The results of this study showed that no cases of severe or clinically significant long-term and short-term adverse events, including injection toxicity and abnormal tissue formation in patients, were recorded. This study also showed that intrabronchial injection of adipose-derived autologous stromal cells is safe in these patients [127]. Campo and colleagues used bone marrow-derived mesenchymal stem cells in mild to moderate pulmonary fibrosis patients. In this study, 10 × 10^6^, 50 × 10^6^, and 100 × 10^6^ cells were injected intrabronchially into 13 patients as a single dose. The results showed that endobronchial infusion of BM-MSCs did not cause serious side effects in patients, but a related proportion of patients progressed clinically. Autologous use of MSCs for three patients appears to be troublesome due to MSC BM-BM genomic instability [128]. In another study, MSC cells in 9 patients with pulmonary fibrosis were injected intravenously with 20, 100, or 200 10^6^ cells as a single dose. The results of this trial showed that human mesenchymal stem cell injections are safe in patients with IPF [129]. CHAMBERS et al. used placental-derived mesenchymal stem cells in 8 patients with IPF. In four patients, 1 × 10^6^ and four patients, 2 × 10^6^ cells were injected intravenously, and patients were monitored for six months. This study showed that intravenous injection of these cells was safe, and there was no evidence of worsening fibrosis [111]. Placenta-derived mesenchymal stem cells have received more attention today than other sources due to their ease of isolation and proliferation. In another study by Averyanov et al., 20 patients with idiopathic pulmonary fibrosis with reduced pulmonary function were selected. MSC cells were injected intravenously with 2 × 10^8^ cells in two doses every three months. In these patients, no serious side effects were observed with the injection of cumulative doses of MSC. Lung function improved during the treatment period in these patients, and DLCO, FVC, and 6MWD parameters showed significant improvement [130].

In the meantime, clinical trial studies using mesenchymal stem cells in pulmonary fibrosis have been recorded at https://clinicaltrials.gov/, which is shown in Table 2.

**Table 2:** Clinical trials in MCS therapy on pulmonary fibrosis

NO	Title and sponsor	Trial ID	Source of MSC	location	Design	Primary outcome	Recruitment status	Phase
1.	Safety of Cultured Allogeneic Adult Umbilical Cord Derived Mesenchymal Stem Cell Intravenous Infusion for IPF Sponsor: The Foundation for Orthopaedics and Regenerative Medicine	NCT05016817	UC-MSC	Island in Antigua and Barbuda	Open Label, Interventional, Safety of Cultured Allogeneic Adult Umbilical Cord Derived Mesenchymal Stem Cell Intravenous Infusion for the Treatment of Idiopathic Pulmonary Fibrosis N:20	Safety (adverse events)	Recruiting August 23, 2021	Phase 1
2.	Allogeneic Human Cells (hMSC)in Patients With Idiopathic Pulmonary Fibrosis Via Intravenous Delivery (AETHER) (AETHER) Sponsor: Joshua M Hare	NCT02013700	–	United States, Florida	A Phase I, Randomized, Blinded and Placebo-controlled Trial to Evaluate the Safety, Tolerability, and Potential Efficacy of Allogeneic Human Mesenchymal Stem Cell Infusion in Patients With Idiopathic Pulmonary Fibrosis N: 9	To determine the safety and tolerability of intravenous allo hMSCs in patients with Idiopathic Pulmonary Fibrosis (IPF)	Terminated (Study completed) March 9, 2021	Phase 1
3.	Infusion of Allogeneic Mesenchymal Stem Cells in Patients With Diffuse Cutaneous Systemic Sclerosis With Refractory Pulmonary Involvement Sponsor: Universidad de la Sabana	NCT04432545	WJ-MSC	Colombia	Expanded Access, Infusion of Allogeneic Stromal Mesenchymal Stem Cells From Wharton´s Jelly in Patients With Diffuse Cutaneous Systemic Sclerosis With Refractory Pulmonary Involvement to Treatment	–	Available June 16, 2020	–
4.	A Study on Radiation-induced Pulmonary Fibrosis Treated With Clinical Grade Umbilical Cord Mesenchymal Stem Cells Sponsor: Jianwu Dai	NCT02277145	UC-MSC	China, Chongqing	Interventional, open-label, Phase I Study of Radiation-induced Pulmonary Fibrosis Treated With Clinical Grade Umbilical Cord Mesenchymal Stem Cells N:10	Composite indicators, including quantitative analysis of CT density histograms, self-evaluation and changes of TGF-β1 content Safety Evaluation	Completed July 24, 2019	Phase 1
5.	Safety and Efficacy of Allogeneic Mesenchymal Stem Cells in Patients With Rapidly Progressive Interstitial Lung Disease Sponsor: Federal Research Clinical Center of Federal Medical & Biological Agency, Russia	NCT02594839	BM-MSC	Moscow, Russian Federation	Open Label, Randomized, A Phase I-II Study to Evaluate Safety and Efficacy of Allogeneic Bone-Marrow Mesenchymal Stem Cells in Patients With Rapidly Progressive Interstitial Lung Disease N:20	Safety: Number of serious adverse events	Completed January 9, 2018	Phase 1 Phase 2
6.	Study of Autologous Mesenchymal Stem Cells to Treat Idiopathic Pulmonary Fibrosis (CMM/FPI) Sponsor: Clinica Universidad de Navarra, Universidad de Navarra	NCT01919827	BM-MSC	Salamanca, Spain	Open Label, Interventional, Treatment of Idiopathic Pulmonary Fibrosis With Bone Marrow Derived Mesenchymal Stem Cells N:17	Number of participants with adverse side effects	Completed May 3, 2018	Phase 1
7.	Role of Stem Cell Therapy in Interstitial Pulmonary Fibrosis Sponsor: Assiut University	NCT03187431	BM-MSC	Egypt	Open Label, Interventional, Mesenchymal Stem Cell as Therapeutic Modality in Interstitial Pulmonary Fibrosis N:12	Number of participants with treatment related side effects as infection, allergic reaction, disease acute exacerbation, and ectopic tissue formation	Unknown June 15, 2017	Phase 1
8.	A Study to Evaluate the Potential Role of Mesenchymal Stem Cells in the Treatment of Idiopathic Pulmonary Fibrosis (MSC in IPF) Sponsor: The Prince Charles Hospital	NCT01385644	PD-MSC	Minnesota, United States	Open Label, Single Group Assignment, A Phase I Study to Evaluate the Potential Role of Mesenchymal Stem Cells in the Treatment of Idiopathic Pulmonary Fibrosis N:8	Number of Participants Who Demonstrated Acute Adverse Events Following Infusion	Completed December 29, 2015	Phase 1
9.	Evaluate Safety and Efficacy of Intravenous Autologous ADMSc for Treatment of Idiopathic Pulmonary Fibrosis Sponsor: Kasiak Research Pvt. Ltd	NCT02135380	AD-MSC	India	A Prospective, Multicentric, Phase I/II, Open Label, Randomized, Interventional Study to Evaluate the Safety and Efficacy of Intravenous Autologous Adipose Derived Adult Stem Cells for Treatment of Idiopathic Pulmonary Fibrosis (IPF) N:210	Safety	Unknown May 13, 2014	Phase 1 Phase 2

*UC-MSC* Umbilical cord-derived mesenchymal stem cells, *WJ-MSC* Wharton's jelly-derived mesenchymal stem cells, *BM-MSC* bone marrow-derived mesenchymal stem cell, *PD-MSC* Placenta-derived mesenchymal stem cells, *AD-MSC* adipose tissue-derived mesenchymal stem cell

### Possible disadvantages of using MSCs in clinical trials

The use of new therapies such as cell therapy always has advantages and disadvantages and faces various challenges. One of the critical things in using stem cells is the tumorigenic power of these cells. The risk associated with tumorigenesis after stem cell transplantation is controversial and has been evaluated in various studies. Stem cells, like tumor cells, can proliferate for a long time, have high viability, and are resistant to apoptosis [131]. In general, donor age, recipient tissue, and growth regulators can affect transplanted mesenchymal stem cells [132, 133]. In patients undergoing long-term chemotherapy and radiotherapy, because of the immune system inadequacy, transplantation of these cells increases the risk of tumorigenesis for the patient [134]. In addition, there is a piece of evidence that the appropriate number of these cells does not reach the desired location. An insufficient number of cells reach the injury site due to their short survival and the entrapment of most of these cells after intravenous injection into the lungs [135–137]. Problems such as alloimmunization may result in the re-injection of mesenchymal stem cells, while many studies have suggested that a single MSC injection is safe for the patient and does not stimulate the immune system [138]. In addition, materials used to stimulate the growth and differentiation of these cells in the laboratory environment, including FBS, may elicit an immune response in the patient's body [139].

## Conclusion

Wharton Jelly is a good source for extracting MSCs and applying it in the clinic. These cells have more stemness properties than other tissue-derived mesenchymal stem cells, and there are no ethical problems with using these MSCs. According to preclinical studies on mesenchymal stem cells derived from different sources in the pulmonary fibrosis model, it seems that Wharton jelly-derived cells could be excellent sources for the treatment of this disease in the future. Therefore, it is possible to open a clear perspective in the treatment of pulmonary fibrosis in the future by conducting more clinical trial studies using WJ-MSC. In the meantime, selecting the appropriate cell dose, the number of injections, injection method, precise protocols for isolation, cell culture, and the proliferation of these cells can be the beginning of a new treatment procedure in patients with pulmonary fibrosis in the future.

## Data Availability

Not applicable.

## Abbreviations

PF: Pulmonary fibrosis
IPF: Idiopathic pulmonary fibrosis
ATII: Alveolar cell type 2
MSCs: Mesenchymal stem cells
PD-MSCs: Placenta-derived mesenchymal stem cells
HBM-MSC: Human bone marrow-derived mesenchymal stem cell
UC-MSC: Umbilical cord-derived mesenchymal stem cells
WJ-MSC: Wharton’s jelly-derived mesenchymal stem cells
AD-MSC: Adipose tissue-derived mesenchymal stem cell
HLA: Human leukocyte antigen
VEGF: Vascular endothelial growth factor
IGF-1: Insulin-like growth factor 1
bFGF: Basic fibroblast growth factor
NGF: Nerve growth factor
TGF-b1: Transforming growth beta-1
IFN-γ: Interferon-γ
TNF-α: Tumor necrosis factor α
SFTPC: Surfactant protein C
IL-1α: Interleukin-1 alpha
IL-10: Interleukin 10
IL-1β: Interleukin-1 beta
SDF-1/CXCL12: Stromal cell-derived factor 1
MCP 1/CCL2: Monocyte chemoattractant protein-1
HGF: Hepatocyte growth factor
PGE2: Prostaglandin E2
IDO: Indolamine 2, 3-dioxygenase
NK cell: Natural killer cells
PD-L1: Programmed death ligand 1
MMPs: Matrix metalloproteases
TIMPs: Tissue metalloproteinase inhibitors
EMT: Epithelial mesenchymal transport
CTD-IP: Interstitial pneumonia in connective tissue diseases
UIP-HLF: Usual interstitial pneumonia- Human primary lung fibroblasts
UC: Umbilical cord
VLA-4: Very late antigen 4
JAK: Janus kinase
BLM: Bleomycin
6MWD: 6-Min walk distance
FVC: Forced vital capacity
DLCO: Diffusing capacity of lung for carbon monoxide
